# Primary Hyperparathyroidism is Underdiagnosed and Suboptimally Treated in the Clinical Setting

**DOI:** 10.1007/s00268-018-4574-1

**Published:** 2018-03-12

**Authors:** Jacob Enell, Haytham Bayadsi, Ewa Lundgren, Joakim Hennings

**Affiliations:** 1 Department of Surgical and Perioperative Sciences, Umeå University, 901 85 Umeå, Sweden; 2 Department of Surgical Sciences, Uppsala University, 751 85 Uppsala, Sweden

## Abstract

**Purpose:**

To evaluate whether patients presenting with laboratory results consistent with primary hyperparathyroidism (pHPT) are managed in accordance with guidelines.

**Methods:**

The laboratory database at a hospital in Sweden, serving 127,000 inhabitants, was searched for patients with biochemically determined pHPT. During 2014, a total of 365 patients with biochemical laboratory tests consistent with pHPT were identified. Patients with possible differential diagnoses or other reasons for not being investigated according to international guidelines were excluded after scrutinizing records, after new blood tests, and clinical assessments by endocrine surgeons.

**Results:**

Altogether, 92 patients had been referred to specialists and 82 had not. The latter group had lower serum calcium (median 2.54 mmol/L) and PTH (5.7 pmol/L). Out of these 82 cases, 9 patients were diagnosed with pHPT or had some sort of long-term follow-up planned as outpatients.

**Conclusion:**

Primary hyperparathyroidism is overlooked and underdiagnosed in a number of patients in the clinical setting. It is important to provide local guidelines for the management of patients presenting with mild pHPT to ensure that these patients receive proper evaluation and follow-up according to current research.

## Introduction

Over-production of parathyroid hormone (PTH), or primary hyperparathyroidism (pHPT), is a common endocrine disease [1]. Its prevalence increases with age, and women are affected more often than men [2]. Studies on adult Swedish populations have demonstrated a prevalence of 0.22–0.36% [1] and exceeding 3% [3, 4] in postmenopausal women.

The most common cause of the disease is a single adenoma, even though some patients have multiple adenomas or hyperplasia of all glands [5]. Parathyroid carcinoma is uncommon and causes <1% of cases of parathyroid diseases [5–7].

Histological change decreases the parathyroid gland’s sensitivity to calcium concentration. This results in impaired inhibition of PTH secretion. High levels of PTH in turn increase the serum concentration of calcium. This biochemical imbalance between serum calcium levels and PTH is the basis for diagnosis. It is important to recognize that pHPT might be present even when calcium and PTH are within normal ranges [8, 9].

There are a number of differential diagnoses that can cause a similar imbalance between calcium and PTH. Secondary hyperparathyroidism (sHPT) is the most common and is caused by renal failure or vitamin D deficiency [10]. Some drugs, e.g., lithium and thiazides, can simulate the biochemical imbalance of pHPT [11, 12]. Familial hypocalciuric hypercalcaemia (FHH) is an uncommon and benign condition with defects in the calcium-sensing receptor, causing a reduced secretion of calcium in urine, elevated serum calcium levels and inadequately depressed PTH levels. Apart from family history and genetic testing, FHH is confirmed by measuring total urine calcium over a 24-h period [13].

Several organs might be damaged by the disease. Patients affected by pHPT have an increased risk of cardiovascular diseases [14, 15]. Deteriorated lipid metabolism, endothelial dysfunction, hypertension and left ventrical hypertrophy seem to be factors mediating the increased cardiovascular risk [14, 16]. Other complications include nephrolithiasis [17], nephrocalcinosis [17] and osteoporosis [18]. Psychiatric problems such as fatigue and depression, decreasing the perceived quality of life, are common but unspecific complaints. Nevertheless, the frequency of these symptoms is elevated in patients with pHPT and some studies have shown a positive effect from the surgical treatment of pHPT [19, 20].

Biochemical testing of calcium and PTH is easily accessible across most of the world, and this contributes to an increased prevalence of patients diagnosed with biochemically mild pHPT. However, a biochemically mild profile does not guarantee mild symptoms. A large portion of patients who present with biochemically mild pHPT have symptoms at presentation [8, 9], if these are properly searched for. It is still not clear which patients with mild and overtly asymptomatic pHPT will benefit from surgery, but some conservatively treated patients will develop a loss of cortical bone, progressive hypercalcaemia [21] and neuropsychiatric symptoms [22].

The only definitive treatment is surgical removal of the diseased gland(s). According to the Scandinavian Quality Register for Thyroid, Parathyroid and Adrenal Surgery in 2014, 94% of patients are cured (defined as normalized serum levels of calcium and PTH) after undergoing a parathyroidectomy [23]. Despite the low risk of complications with surgery [23], the often benign progress of pHPT makes it essential to carefully evaluate the risk profile of each individual before deciding to whom a parathyroidectomy should be offered. After differential diagnoses are excluded, information on the patient’s symptoms, overt organ damage, comorbidity, current medications and age should be considered [24].

In Sweden, no firm guidelines exist on how to evaluate patients presenting with biochemically mild and overtly asymptomatic pHPT. The number of patients seen at the departments of endocrinology or endocrine surgery is not consistent with the presented prevalence of pHPT. This suggests that some patients with biochemically determined pHPT have not been evaluated by a specialist in endocrine surgery or endocrinology. Because of the lack of guidelines, these patients risk being incorrectly evaluated and followed up. If these patients do exist, their characteristics and symptoms, as well as the best way to handle these patients, have not yet been studied in a Swedish setting.

To conclude, the aim of this study was to investigate how patients presenting with laboratory results consistent with pHPT are managed in respect to referral to specialists and follow-up in a setting without firm local guidelines.

## Materials and methods

### Database of laboratory results

The criteria for biochemically determined pHPT were defined as albumin-corrected serum calcium ≥ 2.40 mmol/L (ref 2.15–2.50 mmol/L), serum PTH ≥ 4.3 pmol/L (ref 1.6–6.9 pmol/L) and serum creatinine < 175 mmol/L (ref 50–90 mmol/L) in the same test. PTH was analysed in a Cobase 601 using a third-generation PTH (PTH STAT/PTH intact, Roche Diagnostics GmbH) assay.

**Fig. 1:**
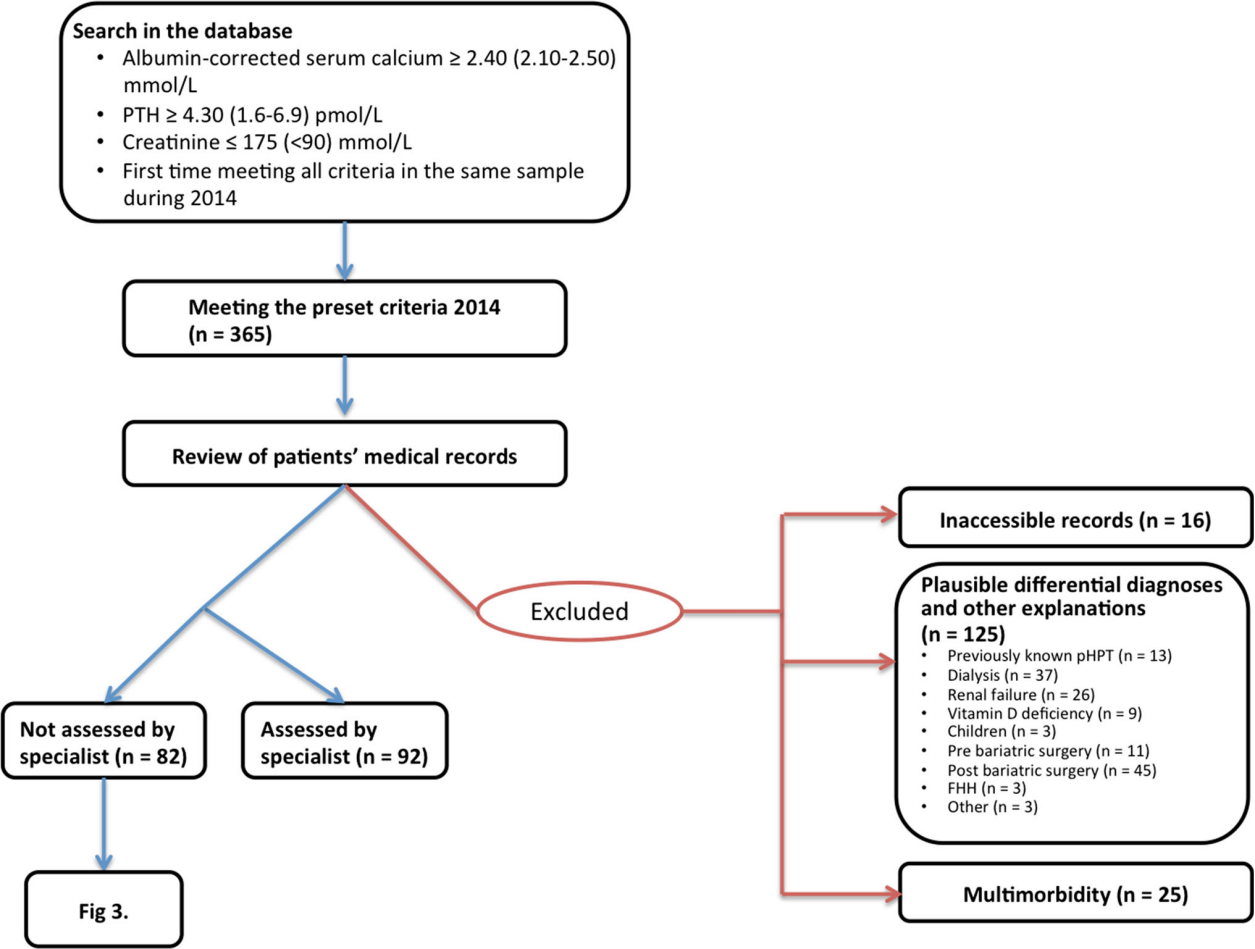
In total, 365 patients presenting with preset criteria for possible pHPT for the first time during 2014. Patients with differential diagnoses or other explanations for the imbalance in their calcium homeostasis were excluded after scrutinizing their patient records

From 12 January 2011 to 24 November 2015, a total of 1828 individuals fulfilling these criteria in the same blood sample for the first time were identified in the database of laboratory results at Östersund Hospital in northern Sweden, which serves 127,000 inhabitants in the region Jämtland-Härjedalen. The data were obtained from all the primary healthcare centres of the region as well as all departments in the hospital. The 365 individuals tested during 2014 were examined further (Fig. 1).

### Review of records

The patients’ records were scrutinized concerning further handling and to exclude patients with plausible differential diagnoses (Fig. 1). Altogether, 16 patients had to be excluded due to inaccessible medical records. The records of the remaining patients were reviewed at least 1 year after the initial blood tests. At this point, 92 patients were referred to a specialist, or the physician primarily ordering the tests was classified as a specialist (endocrinologist, endocrine surgeon or nephrologist). These cases were thereby assumed to have been properly attended to and were excluded. Thirteen patients had previously been diagnosed with pHPT and were also excluded.

Patients (*n* = 37) with tests ordered by the dialysis clinic were assumed to have sHPT and were excluded, as were 26 patients due to the high probability of renal sHPT. The majority of them (*n* = 22) had either progressively elevating levels of creatinine or previously diagnosed chronic renal failure, although with creatinine levels below 175 mmol/L in this test. Two children with chronic renal failure and two patients with a single kidney met the criteria, but were all excluded because of plausible sHPT. An additional three children were excluded due to the low probability of pHPT at such an early age. A 1-year-old boy had hypercalcaemia (2.56 mmol/L) in combination with an infection with preserved PTH (5.0 pmol/L). A girl of the same age was investigated because of diverging from the normal growth curve and was found to have hypercalcaemia (2.64 mmol/L) and preserved PTH, plus elevated serum alkaline phosphatase. An older girl (6 years old) presented with unspecific neurological symptoms and had mild hypercalcaemia (2.51 mmol/L), but her PTH (4.8 pmol/L) was within the normal reference range.

In total, nine cases were considered as suffering from vitamin D deficiency and were excluded; five had low vitamin D levels (<50 mmol/L), and four were prescribed vitamin D substitution and their calcium was later normalized.

Patients on waiting lists for bariatric surgery (*n* = 11) and patients with a history of bariatric surgery within 5 years (*n* = 45) were excluded since studies have demonstrated a shift in calcium homeostasis in these groups [25, 26].

Three patients had low total 24-h urine calcium consistent with FHH (1.04–1.97 mmol/day (normal range 2.5–8 mmol/day) and were excluded without further investigations.

Patients’ medication lists were not available in all records, but two patients had to be excluded due to medication that could mimic pHPT. One patient was on lithium and one was treated with calcium combined with vitamin D substitution and bisphosphonates. When the treatment of the latter patient was cancelled, both calcium and PTH were normalized. One patient was suspected to suffer from hyperthyroidism and was excluded for this reason.

Finally, in 25 cases the physician was assumed to have refrained from further investigation due to patient age and comorbidity. At the end of the review, 82 patients with a biochemically established diagnosis of pHPT had not been properly evaluated or followed up.

### Ethics and informed consent

The study was approved by Umeå Universit*y’s* Ethics Committee. Informed consent was collected from all 82 patients who were included in the survey, given new blood testing and assessed by a specialist.

### Statistics

IBM SPSS version 24 was used to calculate mean and median values. Values were not normally distributed. The Mann–Whitney *U* test was used as test of significance, and *p* < 0.05 was considered significant.

## Results

A cohort of 82 patients was identified, all with biochemically determined pHPT according to preset criteria and with no other explanation for the disturbed calcium homeostasis. Of these 82 patients, 9 had been diagnosed with pHPT and/or had monitoring planned in a primary care centre.

**Fig. 2:**
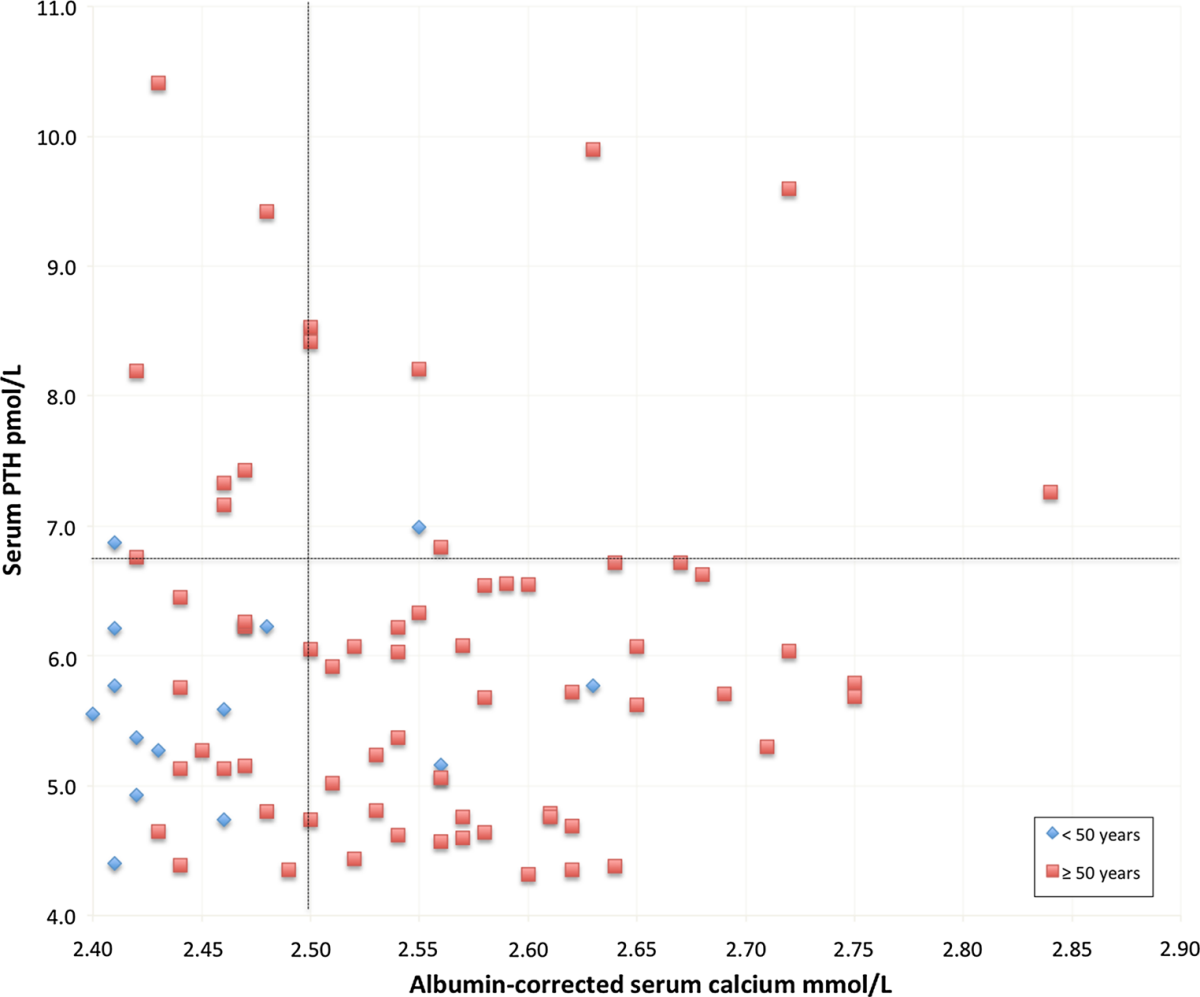
Distribution of albumin-corrected serum calcium and PTH in 82 patients with biochemically determined primary hyperparathyroidism identified in a search of the laboratory results database at Östersund Hospital. Data were collected during 2014, and none of the patients had been assessed by a specialist. The cases are sorted by age. The black dotted lines represent the upper limits of the normal range of albumin-corrected serum calcium (=2.50 mmol/L) and serum PTH (=6.9 pmol/L)

Table 1 presents data from the cohort. Figure 2 plots the distribution of albumin-corrected serum calcium and serum PTH in the 82-patient cohort. Five patients had both serum PTH and albumin-corrected serum calcium above the reference intervals. Conversely, a total of 27 patients had both PTH and albumin-corrected serum calcium within the reference ranges. Eight cases were normocalcaemic with high serum PTH, while 42 individuals were hypercalcaemic with PTH within the reference interval.

**Table 1:** Data from 82 patients with biochemically determined primary hyperparathyroidism identified in a search of the laboratory results database at Östersund Hospital

	Mean	Median	Range	References
Albumin-corrected serum calcium (mmol/L)	2.54	2.54	2.40–2.84	2.15–2.50
PTH (pmol/L)	6.0	5.7	4.3–10.4	1.6–6.9
Creatinine (mmol/L)	73	71	28–132	50–90
Age (years)	63	64	17–85	

	Female	Male
Gender	66 (80%)	16 (20%)

Data were collected during 2014, and none of the patients had been assessed by specialist

Patients who were referred to or primarily managed by a specialist (*n* = 92; data in Table 2) showed significantly higher levels of albumin-corrected serum calcium and serum PTH, as shown in Table 3. Groups were equal regarding gender and median age.

**Table 2:** Data from 92 patients with biochemically determined primary hyperparathyroidism identified in a search of the laboratory results database at Östersund Hospital

	Mean	Median	Range	References
Albumin-corrected serum calcium (mmol/L)	2.65	2.65	2.40–3.17	2.15–2.50
PTH (pmol/L)	8.6	7.4	4.4–26.5	1.6–6.9
Creatinine (mmol/L)	73	69	41–137	50–90
Age (years)	66	68	34–95	

	Female	Male
Gender	74 (80%)	18 (20%)

Data were collected during 2014, and all patients had been referred to or primarily managed by a specialist

**Table 3:** Median laboratory values in two groups of patients with biochemically determined pHPT identified in a search of the laboratory results database at Östersund Hospital

	Assessed by specialist (*n* = 92)	Not assessed by specialist (*n* = 82)	*P* value
Albumin-corrected serum calcium (mmol/L)	2.65	2.54	0.000001
PTH (pmol/L)	7.4	5.7	0.000001
Creatinine (mmol/L)	69	71	0.5
Age (years)	68	64	0.1

Patients who had been assessed by a specialist were compared with those who had not been evaluated. Test of significance: Mann–Whitney *U* test

**Fig. 3:**
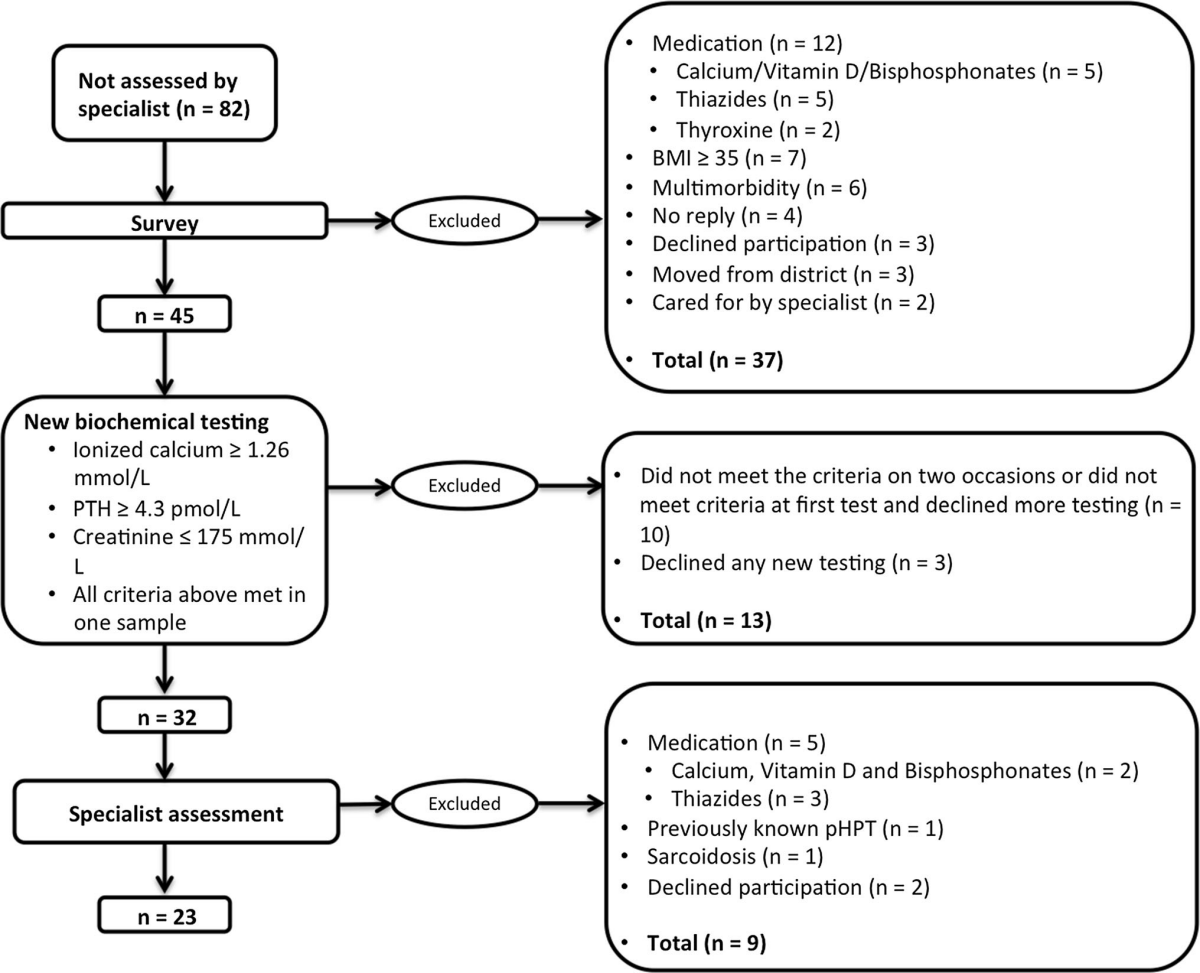
A total of 82 patients with plausible pHPT were not referred to a specialist. Cases with differential diagnoses or other explanations for the imbalance in calcium homeostasis were excluded stepwise after answering a survey and based on results of new blood tests and an assessment by an experienced endocrine surgeon

The 82 patients not assessed by a specialist were invited to answer a survey investigating current and previous medication, comorbidity and body mass index (Fig. 3). From this survey, 12 patients with medication (thiazides, calcium, vitamin D and bisphosphonates) that could possibly influence serum calcium and PTH levels were identified and excluded, as well as seven patients with BMI ≥ 30. Moreover, two individuals had died and four cases were considered too severely ill with multiple diseases to be suitable for treatment or further investigation of their hyperparathyroidism. Three patients declined further participation; four patients did not reply to the invitation, and three patients had moved to other parts of the country and were lost to follow-up. Another two patients had been diagnosed with pHPT and consequently cared for. All these cases were excluded from the study cohort.

A second sampling of serum PTH, ionized calcium and creatinine was arranged for the remaining cohort of 45 patients. Ionized calcium was chosen for these tests to provide a more specific diagnosis of pHPT in a low-range hypercalcaemia [27] as compared to the formerly used albumin-corrected serum calcium values. Limits for exclusion were set at the same levels for PTH (≥4.3 pmol/L) and creatinine (<175 mmol/L) as in the first inclusion criteria, and the limit for ionized calcium was set at 1.26 mmol/L (ref 1.13–1.30 mmol/L), corresponding to an albumin-corrected calcium of 2.40 mmol/L. All criteria had to be met before patients continued to be included in the study. Four patients declined further participation. Patients not fulfilling the criteria after this new blood sample (*n* = 12) were offered one repeated testing. Ten patients did not meet the criteria this second time or declined further samplings and had to be excluded. If they fulfilled the criteria at this second new sampling, they were included in the study (*n* = 2).

Patients in the final study cohort (*n* = 32) were given outpatient appointments at the Endocrine Surgery Unit of the Department of Surgery, Östersund Hospital. At this point, two patients declined further participation in the study. The remaining 30 patients were assessed by one of two experienced endocrine surgeons focussing biochemistry, differential diagnoses and symptoms. Patients on medications able to influence serum calcium and PTH (thiazides *n* = 3, calcium *n* = 2), with previously known pHPT (*n* = 1) and a differential diagnosis (*n* = 1, sarcoidosis), were excluded.

After completing the survey, new blood tests and assessment by an endocrine surgeon (Fig. 2), 23 patients remained in the cohort, with a persistent biochemical pHPT and lack of known differential diagnoses. Table 4 presents laboratory values from these patients’ new blood tests.

**Table 4:** Data from 23 patients diagnosed with pHPT by an endocrine surgeon, 2016/2017

	Mean	Median	Range	References
Ionized calcium (mmol/L)	1.30	1.30	1.26–1.37	1.10–1.30
PTH (pmol/L)	6.4	5.9	4.3–13	1.6–6.9
Creatinine (mmol/L)	74	74	57–104	50–90
Age (years)	62	64	29–82	
Vitamin D (nmol/L)*	60	56	35–107	>0

	Female	Male
Gender	19 (83%)	4 (17%)

The cohort derived from a search of the laboratory results database at Östersund Hospital for patients presenting with presumed pHPT during 2014 who had not been evaluated by a specialist

*Analysed in 15 of the 23 patients

## Discussion

A surprisingly large portion (*n* = 82) of the 365 patients with biochemically determined pHPT were not referred to specialists, despite the lack of differential diagnosis or other reasons to exclude the patient from treatment. However, eleven patients had a long-term plan of follow-up or were diagnosed with pHPT by the physician ordering the initial blood tests. A total of 42 patients underwent new blood sampling, and 32 of them still had an imbalance of calcium and PTH. Ultimately, 23 patients (28% of those not referred to a specialist) were formally diagnosed with pHPT by an endocrine surgeon.

It is important to remember that patients were excluded stepwise without further investigation. Thus, the number of patients with established pHPT but without proper medical management might be higher. Patients with mild pHPT will be subjected to further characterization regarding symptoms and complications due to pHPT, and the results will be published in a forthcoming report.

Mild pHPT is often overtly asymptomatic but can cause silent organ damage which progresses over time. It is known that some patients will suffer progressive hypercalcaemia or organ damage, but, even today, there is no way of predicting which patients with biochemically mild pHPT will benefit from surgery [21]. Therefore, it is important to evaluate every indication for treatment, even in patients with mild pHPT. A study in a North American specialist clinic reported the same frequency of nephrolithiasis in normocalcaemic patients as in those with hypercalcaemia. However, bone density was higher in the normocalcaemic patients [28]. In a group consisting of 52 patients with pHPT, but not meeting any indications for treatment at baseline, 27% of conservatively managed patients developed at least one indication for surgery over a 10-year period [29]. Surgery also had the same effect on normalizing serum calcium and PTH as well as on self-reported symptoms, and this was regardless of whether patients were normocalcaemic or hypercalcaemic before surgery [30]. One study reported that the effect of parathyroidectomy on quality of life in patients with mild pHPT was at least as good as in hypercalcaemic patients [31]. Based on these facts, international guidelines recommend careful investigation of possible organ damage and symptoms after considering differential diagnoses and establishing the diagnosis of pHPT. Selected cases should be offered surgery, and to those not meeting indications for treatment, regular checks of serum calcium and development of organ damage [24] should be offered [32–34]. In the final cohort of 23 patients, surprisingly few were overtly asymptomatic when evaluated with a simple questionnaire. Altogether, 19 patients reported malaise, weakness or reduced power of concentration. One patient suffered from nephrolithiasis and another patient reported osteoporosis.

We can only speculate on the consequences of the delay to potential treatment. However, it is reasonable to assume that a minor group of patients will deteriorate in their biochemical profile [21] and would have benefitted from an early diagnosis, proper monitoring and treatment. For the majority of cases, reduced bone mineral density, the risk of cardiovascular disease and other symptoms of pHPT as, e.g., nephrocalcinosis, also could be assumed to worsen over time.

The reason why the calcium and PTH analyses were ordered was sparsely reported. However, calcium analysis is part of a routine package of analyses available to physicians and is frequently ordered when a screening of the patients’ renal function and electrolyte levels is demanded. In most cases, an incidental finding of hypercalcemia seems to have been the reason for the analysis of PTH.

At the Östersund Hospital, and in most Swedish laboratories, normal values of albumin-corrected serum calcium range from 2.15 to 2.50 mmol/L and PTH from 1.9 to 6.9 pmol/L. The true ratio between PTH and calcium that correlates with histologically proven pHPT is still to be established. There are studies including normocalcaemic cases (calcium ≥ 2.1 mmol/L and PTH ≥ 6.9 pmol/L, [8]) as well as normohormonal cases (calcium ≥ 2.78 mmol/L and PTH < 4.2 pmol/L, [9]). Therefore, it seems reasonable to assume that a significant disturbance of calcium homeostasis exists when calcium levels are in the upper part of the normal range together with non-suppressed PTH levels.

To reduce the number of false positive pHPT patients in the final cohort, we have tried to exclude all patients with a plausible other cause of the disturbed balance of serum calcium and PTH. In total, 156 patients were excluded due to possible differential diagnoses during the different steps of exclusion. A suspicion of a differential diagnosis derived from the patients’ records or list of medications was deemed reason enough for exclusion. Of course, this approach causes a high risk of excluding patients with mild pHPT. On the other hand, false positive cases are more easily eliminated. The 82 patients not being cared for properly were all asked to voluntarily participate in the coming studies. Only 7 patients declined further participation.

The most common differential diagnosis was sHPT due to renal failure. This was expected, as renal failure is a common disorder and the prevalence of sHPT increases with the degree of kidney failure [35]. All cases with serum creatinine > 175 mmol/L and all patients from the dialysis clinic were excluded. The records of those who passed these gateways, but had a history of renal failure, were scrutinized further, and patients were excluded if the probability of sHPT was considered high.

Most of the patients studied did not have vitamin D values analysed, but those who had values <50 mmol/L were also excluded. Values below this level are possibly associated with sHPT [36]. When vitamin D subsequently was analysed in 15 patients in the final cohort, six of them had values below 50 nmol/L, proving the importance of excluding sHPT in this group. Some patients had received vitamin D substitution (even though no analysis of vitamin D levels could be found in their records). If, when followed up, the calcium and PTH values had normalized, these cases were also excluded. Obese patients were excluded, too, as they often suffer from vitamin D deficiency and sHPT, as were those with a history of bariatric surgery [25, 26]. A few patients had their urinary calcium tested, and those with low values were excluded even if the diagnosis of FHH was not confirmed with a family history or genetic testing. Hyperthyroidism is associated with mild hypercalcaemia [37], and even though a preserved PTH level was not expected, one patient with hyperthyroidism was excluded due to this reason.

Moreover, because pHPT in children is so rare, all children (age < 15 years, *n* = 3) were excluded, even though no other completely convincing reason for the disturbed balance between calcium and PTH could be found in their records.

Finally, patients with a record of severe illnesses were excluded. If no severe symptoms or signs of pHPT are present, these patients are normally excluded from surgical treatment and follow-up and the guidelines do not apply to them.

The exclusion process consisted of reviewing records, medications and BMI, and repeating tests of serum calcium, PTH and creatinine levels. In the final cohort, there might still be some falsely diagnosed patients with pHPT, since no analyses of vitamin D values or total urinary calcium levels were performed. On the other hand, no further investigations of the excluded patients have been done and it is reasonable to assume that several of the excluded patients still have pHPT. For example, obesity does not exclude pHPT, both diseases being quite common.

## Conclusion

Patients with biochemically determined, mild pHPT are not investigated or followed up properly. The high proportion of patients presenting with biochemically determined, mild pHPT underlines the importance of providing local instructions to ensure that patients are investigated and followed up according to the most recent international guidelines.
